# Changing Perspectives on the Role of DnaA-ATP in Orisome Function and Timing Regulation

**DOI:** 10.3389/fmicb.2019.02009

**Published:** 2019-08-29

**Authors:** Alan C. Leonard, Prassanna Rao, Rohit P. Kadam, Julia E. Grimwade

**Affiliations:** ^1^Laboratory of Microbial Genetics, Department of Biomedical and Chemical Engineering and Science, Florida Institute of Technology, Melbourne, FL, United States; ^2^Department of Biochemistry, Vanderbilt University School of Medicine, Nashville, TN, United States

**Keywords:** *oriC*, DnaA, DNA replication, replication origin, orisomes, pre-replication complexes, DNA binding proteins, cell cycle

## Abstract

Bacteria, like all cells, must precisely duplicate their genomes before they divide. Regulation of this critical process focuses on forming a pre-replicative nucleoprotein complex, termed the orisome. Orisomes perform two essential mechanical tasks that configure the unique chromosomal replication origin, *oriC* to start a new round of chromosome replication: (1) unwinding origin DNA and (2) assisting with loading of the replicative DNA helicase on exposed single strands. In *Escherichia coli*, a necessary orisome component is the ATP-bound form of the bacterial initiator protein, DnaA. DnaA-ATP differs from DnaA-ADP in its ability to oligomerize into helical filaments, and in its ability to access a subset of low affinity recognition sites in the *E. coli* replication origin. The helical filaments have been proposed to play a role in both of the key mechanical tasks, but recent studies raise new questions about whether they are mandatory for orisome activity. It was recently shown that a version of *E. coli oriC* (*oriC*^*allADP*^), whose multiple low affinity DnaA recognition sites bind DnaA-ATP and DnaA-ADP similarly, was fully occupied and unwound by DnaA-ADP *in vitro*, and *in vivo* suppressed the lethality of DnaA mutants defective in ATP binding and ATP-specific oligomerization. However, despite their functional equivalency, orisomes assembled on *oriC*^*allADP*^ were unable to trigger chromosome replication at the correct cell cycle time and displayed a hyper-initiation phenotype. Here we present a new perspective on DnaA-ATP, and suggest that in *E. coli*, DnaA-ATP is not required for mechanical functions, but rather is needed for site recognition and occupation, so that initiation timing is coupled to DnaA-ATP levels. We also discuss how other bacterial types may utilize DnaA-ATP and DnaA-ADP, and whether the high diversity of replication origins in the bacterial world reflects different regulatory strategies for how DnaA-ATP is used to control orisome assembly.

## Introduction

The molecular mechanism responsible for triggering new rounds of chromosome replication in bacteria is precisely regulated. New replication forks are initiated from a fixed chromosomal site (*oriC*) only once during each cell division cycle and at a time that is compatible with the cellular growth rate (Cooper and Helmstetter, 1968; Skarstad et al., 1986; Boye et al., 1996; Boye et al., 2000; Leonard and Méchali, 2013). The molecular machine responsible for unwinding origin DNA and loading the replicative helicase on exposed single strands (termed the orisome) is assembled at *oriC* and comprises multiple copies of the initiator protein, DnaA (Leonard and Grimwade, 2015), whose activity is regulated by binding to ATP (Sekimizu et al., 1987; Katayama et al., 2017). In *E. coli*, the cellular level of DnaA-ATP fluctuates during the cell cycle (Kurokawa et al., 1999), and the reproducibility of initiation timing from one cell cycle to the next is achieved by coupling orisome assembly to DnaA-ATP levels. This is accomplished via a set of specifically arranged low affinity DnaA-ATP recognition sites in *E. coli oriC* that direct orisome assembly by guiding cooperative binding of the initiator (Zawilak-Pawlik et al., 2005; Rozgaja et al., 2011; described in more detail below).

Following each new round of DNA synthesis, several mechanisms are used by bacteria to restrict inappropriate orisome reassembly, reviewed in Nielsen and Løbner-Olesen (2008), Katayama et al. (2010), and Skarstad and Katayama (2013). The predominant regulatory mechanism used in *E. coli* involves hydrolytic conversion of DnaA-ATP into DnaA-ADP by a replication fork-associated process termed Regulatory Inactivation of DnaA (RIDA) (Katayama and Sekimizu, 1999), which causes rapid hydrolysis of DnaA-ATP shortly after initiation (Kurokawa et al., 1999). The DnaA-ADP that is generated cannot reassemble into active orisomes for two reasons. First, it does not readily interact with all of the low affinity recognition sites in *oriC* (McGarry et al., 2004; Kawakami et al., 2005; Grimwade et al., 2018) (see below). Second, unlike the ATP-bound form, DnaA-ADP is unable to form the oligomeric filaments that are essential for binding to ssDNA, a function that is proposed to mediate both origin unwinding and helicase loading (Erzberger et al., 2006; Duderstadt et al., 2010).

Our main goal for this review is to raise questions about DnaA-ATP’s exclusive role as the active initiator form, based on recent findings demonstrating that DnaA-ADP was active in unwinding a synthetic version of *E. coli oriC* (*oriC*^*allADP*^) that allows both DnaA-ATP and DnaA-ADP to access all recognition sites (Grimwade et al., 2018). Chromosomal *oriC*^*allADP*^ was also activated *in vivo* by mutant DnaAs that were defective in adenine nucleotide binding or ATP-dependent oligomerization. However, although functional orisomes were formed on *oriC*^*allADP*^, they were unable to trigger properly timed initiation events, revealing that the observed mechanical activity of DnaA-ADP is separate and distinct from the DnaA-ATP-dependent role as a timing regulator. In this review, we discuss the implications of these observations, and discuss how the high level of *oriC* nucleotide sequence diversity among bacterial types may result in orisome assembly pathways that use one or both nucleotide forms for mechanical functions, while reserving the role of DnaA-ATP as a regulator of initiation timing.

## Origin Recognition by Dnaa

**FIGURE 1:**
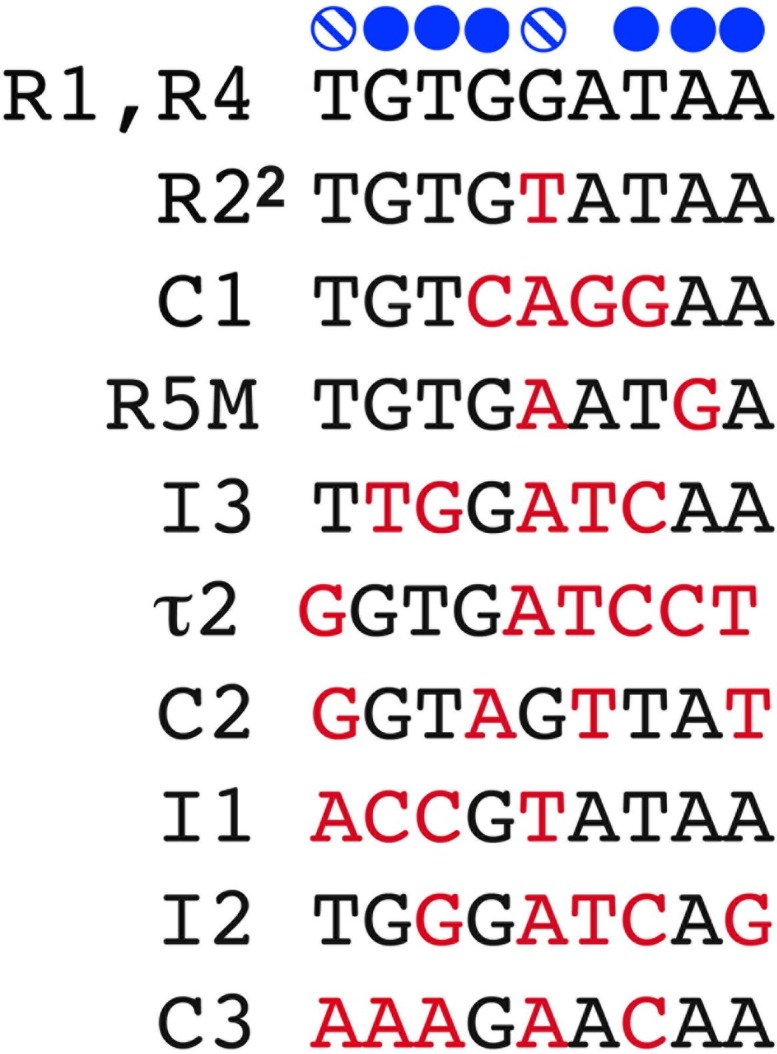
DnaA recognition site sequences in *E. coli* oriC. The 9 mer recognition sequences of the 11 DnaA recognition sites are shown. Bases marked in red deviate from the consensus (shown at top). Solid blue circles mark regions where DnaA makes base-specific contacts on one of the two DNA strands, and the hatched blue circles mark where DnaA makes Van der Waals contacts with thymidine, if present.

Almost all bacterial replication origins contain clusters of the 9 bp sequence 5′-TGTGGATAA-3′ (termed the R box) which is the consensus sequence for DnaA recognition. In *E. coli oriC*, there are two R boxes (R1 and R4) that perfectly match the consensus sequence, and one box (R2) that deviates from consensus by one bp (Figure 1); these three sites bind both DnaA-ATP and DnaA-ADP with high affinity (k_*d*_ = 4–20 nM) (Sekimizu et al., 1987; Schaper and Messer, 1995). Amino acid residues in the helix-turn-helix motif in DnaA’s C-terminal domain (IV) make base-specific hydrogen bonds with nucleotides on one of the two strands at positions 2, 3, 4, 7, 8, and 9 of each R box, as well as Van der Waals contacts with the thymidines that may be present in positions 1 and 6 (Erzberger et al., 2002; Fujikawa et al., 2003) (contacts are summarized at the top of Figure 1).

*E. coli oriC* also contains eight less canonical DnaA binding sites, most of which were identified only after *in vitro* DnaA binding assays (Grimwade et al., 2000; Rozgaja et al., 2011). These cryptic sites deviate from the consensus R box sequence by 2 or more bp (Figure 1), which disrupts some base-specific contacts (Figure 1). While these sites bind DnaA specifically (McGarry et al., 2004; Rozgaja et al., 2011), their affinity for the initiator is reduced so that dissociation constants for individual sites cannot be measured (Schaper and Messer, 1995). In fact, none of the identified low affinity sites are able to bind DnaA independently; rather, DnaA must be recruited and positioned for them by nearby bound DnaA (Schaper and Messer, 1995; Rozgaja et al., 2011). Six of the lower affinity sites (τ2, I1, I2, I3, C2, and C3) preferentially bind DnaA-ATP (McGarry et al., 2004; Kawakami et al., 2005; Grimwade et al., 2018), and occupation of these sites also requires physiological levels of ATP (0.5–5 mM) (Saxena et al., 2013), as well as interactions between a critical arginine (R285) in DnaA’s domain III and the bound ATP of an adjacent DnaA molecule (Kawakami et al., 2005)(discussed further below). While it is not known why these six sites prefer DnaA-ATP, it is probable that conformational differences between DnaA-ATP and DnaA-ADP play a role. The amino acids involved in ATP/ADP binding and hydrolysis are located in a central domain of DnaA (domain III) adjacent to the DNA binding domain (domain IV) (Erzberger et al., 2002; Nishida et al., 2002; Iyer et al., 2004). When bound to ATP, domain IV bends toward domain III, bringing amino acids from both domains into proximity (Erzberger et al., 2006). Physiological levels of ATP are also reported to alter DnaA conformation (Saxena et al., 2015). Conformational changes that alter domain III interactions and allow amino acids outside of domain IV to participate in binding should also increase contacts between DnaA and the low affinity DnaA-ATP sites, thereby compensating for the lack of base-specific DnaA/DNA interactions. Comparing the sequences of the DnaA-ATP sites with the R box sequence (Figure 1) suggests that positions 1–4 of the 9 mer binding sites play a greater role in determining preference for DnaA-ATP. It is important to note that not all low affinity sites preferentially bind DnaA-ATP. This is evidenced by the remaining two weak sites in *oriC* (R5M and C1), which were shown by our laboratory to bind both DnaA-ATP and DnaA-ADP (Grimwade et al., 2007, 2018), although there are conflicting reports which show occupation of these sites only by DnaA-ATP (Ozaki et al., 2012). We note that converting the non-discriminatory R5M sequence into the DnaA-ATP-preferring I2 site resulted in delayed initiation *in vivo*, suggesting that R5M is normally occupied by DnaA-ADP (Grimwade et al., 2007).

**FIGURE 2:**
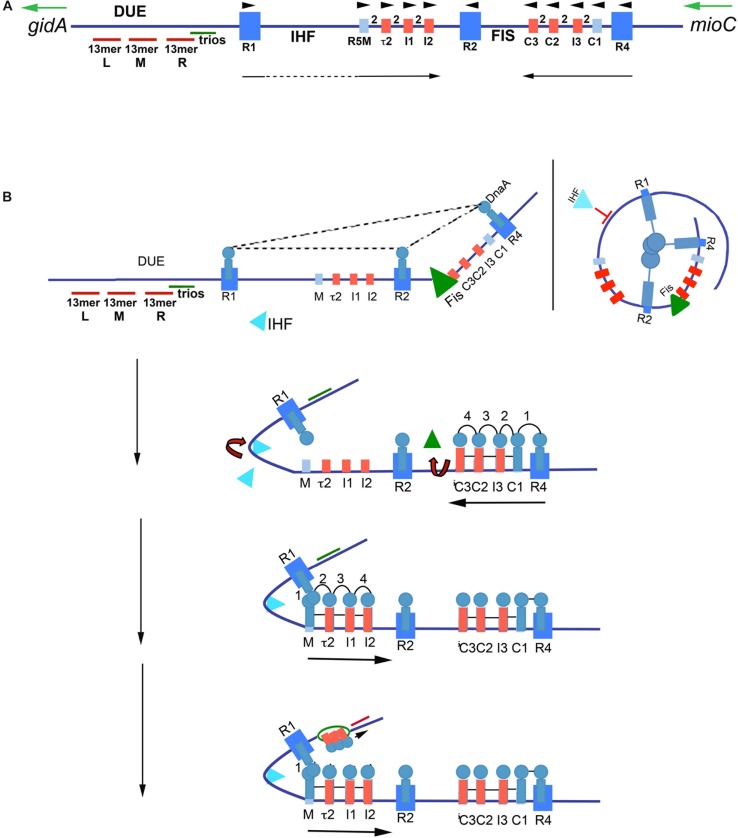
Model of staged orisome assembly. **(A)** Map of *E. coli oriC*. High affinity R boxes are marked by large blue rectangles, low affinity DnaA-ATP recognition sites are marked by small red rectangles, and low affinity non-discriminatory recognition sites are marked by small light blue rectangles. Left (l), middle (M), and right (R) 13 mer AT-rich repeats are shown, as well as the locations of the DnaA-trio elements, and Fis and IHF binding regions. Green arrows mark direction of transcription of flanking genes *gidA* and *mioC*, black arrows mark the direction of DnaA binding progression, and black arrowheads mark the orientation of the recognition sites based on the direction faced by the arginine finger (R285) of bound DnaA. **(B)** Stages of orisome assembly. Stage 1 (left): After initiation of chromosome replication, DnaA rebinds to high affinity R1, R2, and R4 sites. Fis is also bound at this stage, but IHF is not. Low affinity sites are unoccupied. Dashed lines indicate interaction between bound DnaA molecules (right): Looping of DNA to allow bound DnaA molecules to interact. Stage 2: DnaA bound to R4 recruits DnaA for binding to C1. DnaA then progressively fills the remaining arrayed sites, forming an oligomer in the gap region between R2 and R4. The DnaA oligomer displaces Fis, and loss of Fis allows IHF to bind to its cognate site. Stage 3: The bend induced by IHF binding allows DnaA, recruited by R1, to bind to R5M, and form a cross-strand DnaA interaction. A DnaA oligomer then progressively grows toward R2, bound to arrayed low affinity sites, and anchored by R2. Stage 4: *oriC* DNA is unwound in the DUE, and DnaA in the form of a compact filament binds to the ssDNA in DnaA-trios. Figure adapted from Leonard and Grimwade (2015).

All of *E. coli*’s 11 DnaA recognition sites lie to the right of the DNA Unwinding Element (DUE) (Figure 2A). The three high affinity R boxes are spaced such that R1 is immediately left of the DUE, R2 is central, and R4 is located at the right border of the origin (Figure 2A). This widely spaced positioning defines two gap regions where the low affinity sites are located (Rozgaja et al., 2011). Each gap region contains an array of four low affinity sites, each separated from each other by 2 bp (Figure 2A). This specific positioning of *oriC* recognition sites facilitates cooperative DnaA binding, and ordered orisome assembly (Rozgaja et al., 2011; described below).

## Ordered Orisome Assembly

In *E. coli*, orisome assembly begins when DnaA re-binds to the three high affinity R boxes immediately after the initiation of each round of chromosome replication (Nievera et al., 2006). This tightly bound DnaA plays two important roles. The first is to inhibit unscheduled unwinding of *oriC*, since the DUE is a region of intrinsic helical instability and is subject to spontaneous unwinding when *oriC* is unoccupied (Kowalski and Eddy, 1989). DnaA binding to R1, R2, and R4 constrains *E. coli oriC*, eliminating spontaneous unwinding (Kaur et al., 2014). Although details of the constraint mechanism remain unclear, the most likely scenario involves a trimeric complex formed by interactions among the N-terminal, self-oligomerization domains (domain I) (Simmons et al., 2003) of the bound DnaA molecules (Kaur et al., 2014; Figure 2B), perhaps stabilized by the DiaA protein (Ishida et al., 2004). However, domain I-domain I interactions are limited over a distance that is determined by the length of the flexible linker (domain II) that joins each domain I to the rest of the DnaA molecule (Messer et al., 1999; Nozaki and Ogawa, 2008). Therefore, to make the postulated trimeric complex, *oriC* DNA would need to form loops to place the three bound DnaA molecules close enough to interact, similar to those formed in the nucleosomes of eukaryotes (Figure 2B). Alternatively, individual DnaA molecules bound at each R box may be sufficient to clamp the DNA in a way that prevents untwisting without further interactions.

The second role of DnaA binding to R boxes is formation a scaffold that recruits additional DnaA molecules to occupy the adjacent low affinity sites (Miller et al., 2009), and begin the next stage of orisome assembly (Figure 2B). Because this role is analogous to that played by the Origin Recognition Complex (ORC) of eukaryotes (Duncker et al., 2009), the structure formed by DnaA binding to the high affinity sites has been termed the bacterial ORC, or bORC (Nievera et al., 2006). DnaA molecules bound to R1 and R4 recruit additional DnaA using their N-terminal domains, and position it for binding to the nearest low affinity site (R1 to R5M and R4 to C1) (Miller et al., 2009; Figure 2B). DnaA located at R2 does not normally donate DnaA to either of its nearest sites if R1 and R4 are capable of performing this duty (Rozgaja et al., 2011).

Once DnaA is bound to C1 or R5M, the close positioning of low affinity sites promotes cooperative binding of DnaA-ATP to the remaining sites in the right and left arrays, respectively (Figure 2B), progressing from C1 or R5M into the center of *oriC*, toward R2 (Rozgaja et al., 2011). While cooperative binding involves interactions between the domain I regions of donor and recruited DnaA (Rozgaja et al., 2011), domain III regions may also play a role, and the close spacing of the sites is proposed to foster formation of oligomeric DnaA-ATP filaments (Erzberger et al., 2002, 2006; Felczak and Kaguni, 2004; Kawakami et al., 2005). DnaA-ATP oligomers assemble when ATP-associated with DnaA’s domain III in one bound molecule interacts with a critical arginine (R285) in the adjacent molecule. R285 comprises DnaA’s version of the “arginine finger,” a motif that is highly conserved in AAA + (ATPases Associated with various cellular Activities) proteins (Erzberger et al., 2006), with the interaction stabilized by additional amino acid residues (Duderstadt et al., 2010). The orientation of arrayed low affinity binding sites in each half of *oriC* positions bound DnaA-ATP such that their arginine fingers are all facing R2 (Rozgaja et al., 2011; Noguchi et al., 2015). The structures of the two oppositely-oriented DnaA-ATP oligomers have not been solved, but they are presumed to be a more open version of the compact right-handed helical DnaA-ATP filament that has high affinity for single-stranded DNA (Erzberger et al., 2006; Duderstadt et al., 2010).

The 3 bp separation of R4 and C1 allows direct lateral donation of DnaA from a strong to weak site, but the 46 bp distance between R1 and R5M requires DNA bending and cross-strand donation for cooperative binding (Rozgaja et al., 2011). This bend requirement is the basis for a growth rate-regulated switch that ensures synchronous initiations of the multiple copies of *oriC* that obtain during rapid growth conditions (Cooper and Helmstetter, 1968; Roth et al., 1994). During rapid growth, Fis, a growth rate-regulated protein (Nilsson et al., 1992; Mallik et al., 2006), binds to its recognition site between R2 and C3 shortly after the initiation step (Cassler et al., 1995; Figure 2B), during the time period that *oriC* is constrained by DnaA occupying the three high affinity sites (Kaur et al., 2014). The Fis-bound bORC prevents IHF from binding and bending at its cognate site between R1 and R5M (Ryan et al., 2004; Kaur et al., 2014), possibly because the constrained bORC does not allow two bends to be simultaneously placed in *oriC.* The inhibition of bending results in a temporary block of DnaA binding in the left half of *oriC*. As DnaA-ATP levels increase during the cell cycle, progressive DnaA occupation of the right array of sites displaces Fis (Ryan et al., 2004), allowing IHF to bind, resulting in a DNA bend that places R1 sufficiently close to R5M to nucleate filling of *oriC*’s left side low affinity sites (Rozgaja et al., 2011). By acting as a temporary partition between the left and right halves of *oriC* (Gille et al., 1991), Fis is able to delay initiation until the total number of DnaA molecules in the cell exceeds that needed for initiation of a single *oriC* copy; thus, when Fis is finally displaced, all origins in the cell can complete orisome assembly and initiate synchronously (Ryan et al., 2004; Rao et al., 2018). In this way, Fis becomes the primary regulator of initiation timing under rapid growth conditions (Flåtten and Skarstad, 2013). In contrast, during slow growth when *E. coli* carries only one *oriC* copy, Fis levels are too low to occupy *oriC* (Nilsson et al., 1992), and IHF is able to bind and bend the DNA between R1 and R5M, promoting low affinity site occupation in the left region of *oriC* independently of the filling of the right region. In this case, orisome completion and initiation timing is dependent only on the cellular levels of DnaA-ATP being high enough to fill the low affinity DnaA-ATP sites (Rao et al., 2018). At all growth rates, DnaA-ATP occupation of the low affinity sites promotes opening of the DNA duplex in the right region of the DUE (Bramhill and Kornberg, 1988; Grimwade et al., 2000; Figure 2B). However, there is evidence that not all the low affinity sites in *E. coli oriC* are essential for *in vivo* activity (Stepankiw et al., 2009), and *in vitro*, only R5M needs to be occupied by DnaA for unwinding (Sakiyama et al., 2017).

A variety of models have been proposed to explain the mechanism of unwinding (Speck and Messer, 2001; Erzberger et al., 2006; Ozaki et al., 2008; Duderstadt et al., 2011; Ozaki and Katayama, 2012; Zorman et al., 2012), and both the compact and open versions of DnaA-ATP oligomers are implicated in producing the torsional stress required for DNA unwinding. Proposed mechanisms include: an open DnaA-ATP oligomer bound to double-stranded DNA causing formation of right handed supertwists (Erzberger et al., 2006; Zorman et al., 2012); an open DnaA-ATP oligomer bound to double-stranded DNA in the left array of low affinity sites creating a channel that can engage and unwind DUE DNA (Ozaki et al., 2008, 2012) and a compact DnaA-ATP oligomer stretching and unwinding DUE DNA (Duderstadt et al., 2011; Duderstadt and Berger, 2013).

Once unwound, the single-stranded DNA binds to DnaA-ATP, which stabilizes the open structure (Figure 2B) to promote expansion of the initiation bubble and assist with DNA helicase delivery (Yung and Kornberg, 1989; Speck and Messer, 2001). In *Bacillus subtilis*, the additional DnaA-ATP used for this purpose was shown to interact with specialized 3 bp sequence motifs, termed DnaA-trios (Richardson et al., 2016; Figure 2A), and it is proposed that the trio elements are a conserved aspect of replication origins. The two end bases of trios can vary, but the middle nucleotide must be A (Richardson et al., 2016). In many bacterial types, there are seven to ten direct repeats of DnaA-trios between the DUE and the nearest (3′) high affinity DnaA recognition site (Richardson et al., 2016); *E. coli* has one of the shorter arrays, containing only three trios. In addition to the oligomer formed using trio-elements, the DnaA bound to the right half of *oriC* has also been implicated in DNA helicase loading (Ozaki and Katayama, 2012).

## A Predominant Role for *E. coli* Dnaa-Atp Is in Origin Recognition and Regulation of Initiation Timing

Although DnaA-ATP is required for activation of wild type *E. coli oriC in vitro*, it has been known for several decades that at least some of the DnaA in functional *E. coli* orisomes can be in the ADP-bound form (Yung et al., 1990). The recognition sites occupied by DnaA-ADP in these mixed orisomes was never identified, but all of the R boxes, as well as R5M and C1, are obvious candidates. In support of this idea, a clever heterologous DnaA binding assay was recently used to demonstrate that functional orisomes could be built when either R1 or R4 was occupied by DnaA-ADP (Noguchi et al., 2015).

**FIGURE 3:**
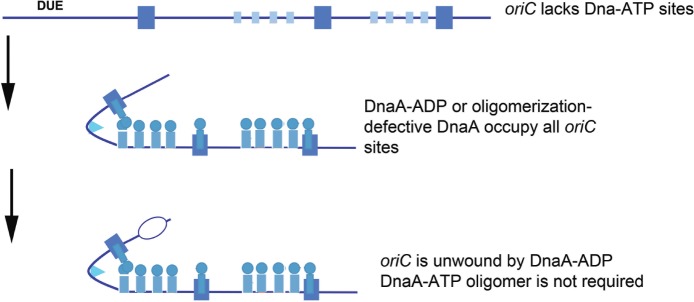
Orisome assembly directed by *oriC*^allADP^. Because *oriC*^allADP^ lacks DnaA-ATP sites, available DnaA-ADP or oligomerization-defective DnaA (shown by blue circles/rectangles) in cells can bind to all sites in the origin. With this form of DnaA, Domain I-domain I interactions can form, but not interactions between the ATP-binding domains. Unwinding is mediated without the formation of oligomeric DnaA filaments. High affinity sites are shown by larger rectangles, and low affinity sites are shown by smaller rectangles.

Regardless of binding locations, the ability to use DnaA-ADP as a component of functional *E. coli* orisomes raises questions about DnaA-ATP as the active form of the initiator. Is DnaA-ATP the active form because it is the only form that can fill all recognition sites, or because it is the only form that can make the higher order oligomeric structures that can perform essential mechanical tasks? To address these issues, a novel version of *oriC* (*oriC*^*allADP*^) was constructed that converted every DnaA-ATP recognition site to one that bound either DnaA-ADP or DnaA-ATP with equivalent low affinities (e.g., each low affinity site was made similar to C1 and R5M) (McGarry et al., 2004; Grimwade et al., 2018). By using *oriC*^*allADP*^, it was possible to examine the activity of orisomes assembled from only DnaA-ADP. Surprisingly, *in vitro*, *oriC*^*allADP*^ plasmids were unwound equally by orisomes assembled with either DnaA-ATP and DnaA-ADP. *In vivo*, use of *oriC*^*allADP*^ as the sole chromosomal replication origin also suppressed the lethality of DnaA mutants with defects in ATP binding and ATP-dependent oligomer formation [DnaA46 and DnaA(R285A), respectively, Grimwade et al., 2018]. Thus, given equal access to *oriC*, both DnaA-ADP and DnaA-ATP are functionally equivalent, with orisomes assembled from either form capable of performing the mechanical actions required to trigger initiation in *E. coli* (Figure 3).

These observations lead to the conclusion that the predominant role for DnaA-ATP in activating wild type *E. coli oriC* must be for origin recognition and site occupation. Since it is normally the case that DnaA-ATP preferentially binds most low affinity sites, initiation timing must be coupled to the availability of this form during the cell cycle. Consistent with this idea, cells triggering chromosome replication from *oriC*^*allADP*^ behaved as if initiation timing was no longer dependent on DnaA-ATP levels. These cells over-initiated, and consequently showed increased sensitivity to replicative stress (Grimwade et al., 2018). Apparently, since DnaA-ADP is not normally degraded in *E. coli*, it was continuously available at levels sufficient to bind to low affinity sites in *oriC*^*allADP*^ and trigger multiple replication rounds. Additional studies, in which only one or two of the DnaA-ATP sites were converted to a version that binds both forms of DnaA equivalently (Rao et al., 2018), revealed that at slow growth rates, each site contributed to the DnaA-ATP regulated initiation timing mechanism. At fast growth rates, Fis, by virtue of its ability to regulate DnaA binding, took over as the major timing regulator, as described above and in Rao et al. (2018). Combined, the data on these synthetic *oriC*s demonstrate that the features of bacterial replication origins involved in mechanical function can be separated from their timing components(s).

The conclusion that DnaA-ADP can activate *E. coli oriC* does not appear to be compatible with models for *E. coli* origin unwinding that invoke assembly of oligomeric DnaA-ATP filaments (see above), although it has yet to be determined whether orisomes made from only DnaA-ADP or DnaA-ATP function in exactly the same way. It is possible that when DnaA-ADP molecules are aligned by binding to arrayed sites, they are capable of forming an unwinding structure similar to one formed by DnaA-ATP, however, if this is the case, the requirement for DnaA-ATP would still be for binding to arrayed sites, not for a unique ability to oligomerize. Alternatively, unwinding mediated by DnaA-ADP might rely on DnaA’s inherent DNA bending activity. DnaA produces a 30–40^∘^ bend in DNA when bound to a 9 mer recognition site (Schaper and Messer, 1995). The concerted bending at multiple sites could provide sufficient stress to unwind the DUE. This mechanism could either replace the need for a DnaA-ATP filament, or it could be used by both DnaA-ATP and DnaA-ADP. If the bending model is correct, then DnaA would produce DNA distortions similar to those caused by binding of archaeal and eukaryotic initiator proteins, generating sufficient torsional stress to unwind the AT-rich DUE (Dueber et al., 2007; Gaudier et al., 2007; Sun et al., 2012).

The observed functionality of DnaA-ADP is also not consistent with mechanisms for unwinding and helicase loading that involve DnaA-ATP filaments associated with DnaA-trios. However, since trio occupation requires DnaA bound to a nearby high affinity R-box (Richardson et al., 2016), and because the trio-proximal R box (R1) is not essential for *E. coli oriC* function (Kaur et al., 2014), it is not known whether DnaA-trios are required in *E. coli*. Thus, *E. coli* may be able to use an alternate mechanism for helicase loading that is not dependent on any unique property of DnaA-ATP.

## Thoughts About the Requirement for Dnaa-Atp in Assembling Orisiomes on Diverse Replication Origin Templates

**FIGURE 4:**
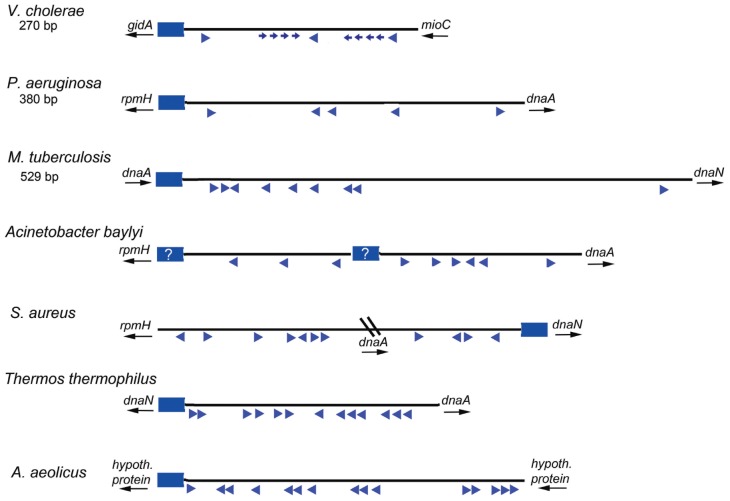
Maps of several bacterial replication origins. Origins from diverse bacteria show many possible configurations. Flanking genes are shown for each origin region, with arrows indicating direction of transcription. The DUE regions for each origin shown by blue rectangles; a question mark is placed if the DUE location is ambiguous. Relative locations of DnaA binding sites are shown by blue arrowheads. Smaller arrows indicate know lower affinity sites. The direction of the arrows indicates the likely orientation of the arginine finger when DnaA is bound.

Based on the studies of *E. coli* orisome assembly, described above, it is clear that the arrangement and nucleotide sequence of DnaA recognition sites in *E. coli oriC* directs ordered orisome assembly, and also couples the cell cycle timing of this process to the availability of DnaA-ATP. Because all other bacterial types must also assemble functional orisomes at the correct cell cycle time, and because DnaA is a highly conserved protein, it is reasonable to expect that the majority of the bacterial *oriC* templates would also be conserved and direct orisome assembly in the same way as *E. coli*. However, this is definitely not the case. A database (DoriC 10.0) containing the nucleotide sequences of thousands of *oriC*s (some putative) reveals enormous diversity among bacterial types, with little overt similarity to most of the features found in *E. coli* other than the presence of multiple R box-type DnaA recognition sites (Luo and Gao, 2019). Figure 4 depicts a few different *oriC* geographies, showing dramatic differences in the number and relative positions of the R-box-like sequences, including both widely separated and closely spaced clusters. However, the variety is far more extensive than can be demonstrated by one figure, and additional details can be found in several papers and reviews (Zawilak-Pawlik et al., 2005; Zakrzewska-Czerwińska et al., 2007; Donczew et al., 2012; Leonard and Méchali, 2013; Wolański et al., 2014; Jaworski et al., 2016). Further, it is likely that cryptic low affinity sites exist in a variety of bacterial origins, but because sequence analysis identifies DnaA binding sites based on their similarity to the consensus R box, DnaA-*oriC* binding assays are required to identify more divergent DnaA recognition sites. Thus, cryptic sites have been mapped in the replication origins of only a few bacterial types other than *E. coli* and its close relatives (Charbon and Lobner-Olesen, 2011; Taylor et al., 2011), and sites similar to the DnaA-ATP sites in *E. coli oriC*’s have not been positively identified in any other bacterial origin.

While we propose that DnaA-ADP might have a greater role than previously believed, it is important to note that a major reason for origin diversity (and the utilization of different forms of DnaA) is that R boxes can be used for functions other than orisome assembly, such as regulating initiation timing (DnaA availability) or for transcriptional regulation. Unlike *E. coli oriC*, which is positioned between the *gidA* and *mioC* genes, many bacterial replication origins are located next to the *dnaA* gene (see Figure 4 for examples). An interesting alternative arrangement in some bacteria places *dnaA* within the interior of *oriC* producing a bi-partite configuration (for examples see *Staphylococcus oriC* in Figure 4 and the *Helicobacter pylori oriC* (Donczew et al., 2012), such that there are clusters of R boxes on either side of *dnaA*. Since the *dnaA* promoter contains DnaA recognition sites used for autoregulation (Atlung et al., 1985; Braun et al., 1985; Ogura et al., 2001), when *dnaA* and *oriC* are adjacent, it is difficult to distinguish R boxes used to regulate *dnaA* expression (by DnaA-ATP and DnaA-ADP) from those used for orisome assembly. Further, some of the R boxes in certain bacterial origin regions may be used to regulate DnaA availability (and initiation timing) by titration (Moriya et al., 1988). In *E. coli*, sites that can titrate DnaA-ATP or DnaA-ADP (Hansen et al., 1991) are located outside of *oriC*, both as individual DnaA recognition sites distributed around the chromosome as well as within a region with high DnaA capacity termed *datA*, located about 460 kb from *oriC* where bound DnaA-ATP is inactivated (Ogawa et al., 2002; Kasho and Katayama, 2013). For some bacteria, *datA*-like sequences may be found in locations proximal to or within *oriC*. It is also possible that all DnaA binding sites in an origin are simply not necessary for functional orisome assembly. For example, in *E. coli*, low affinity sites between R1 and R2 (left side) are implicated in origin unwinding, but the right side sites are not (Stepankiw et al., 2009), although they may play a supportive role in helicase loading (Ozaki and Katayama, 2012). Similarly, only a few of the DnaA boxes in the *B. subtilis* origin, near the DUE, are essential for mechanical functions (Richardson et al., 2019). Recognition sites for regulatory proteins could also contribute to origin diversity. Such regulators would include DNA bending proteins (such as analogs of Fis and IHF) (Brassinga et al., 2002), and proteins which block the interaction of DnaA with their respective recognition sites or suppress cooperative DnaA interactions during orisome assembly. Examples of the latter are described below.

Even after considering regulatory and titration sites, the high variability among bacterial origins raises the obvious conclusion that, although the initiator is conserved, and the essential mechanical functions required for initiation are the same in all bacteria, different assembly paths must be used to form the orisomes that ultimately perform these functions (Jakimowicz et al., 2000; Zawilak-Pawlik et al., 2005). The details of these diverse paths, and how they might utilize DnaA-ATP and DnaA-ADP for mechanical and timing functions remain unanswered questions, but we can speculate about several possibilities.

Since many bacteria carry DnaA-trio sequence motifs located between the DUE and its most proximal R box (Richardson et al., 2016), this feature might play a key role in setting the requirement for DnaA-ATP, or even allowing DnaA-ADP to participate in orisome assembly. Although there is insufficient evidence to determine if they are essential for every bacterial origin, in *B. subtilis* and probably other bacteria, DnaA-trios direct the assembly of critical DnaA-ATP oligomers, and could set the amount of DnaA-ATP required for unwinding and the DNA helicase loading steps (Richardson et al., 2019). In some bacteria, only a small amount of DnaA-ATP may be needed to interact at DnaA-trio elements to effect stable strand separation and/or helicase loading, and the rest of the orisome, including a sub-complex that mediates initial unwinding, could be assembled from DnaA-ATP or DnaA-ADP, depending on the specific origin, as described below.

Other than DnaA-trios, some replication origins appear to lack any recognition sites with preference for DnaA-ATP. This seems to be the configuration of the *oriC*s in *B. subtilis*, *C. crescentus*, and *M. tuberculosis*, among others (Leonard and Méchali, 2013; Wolański et al., 2014). For these origins, the most available form of DnaA in the cell would be used to assemble the orisome, but the active form is expected to be tightly regulated at the level of synthesis and during the inter-initiation interval. Some of the different mechanisms that regulate the availability of DnaA-ATP might also apply to the ADP-bound form if it plays a role in orisome assembly or origin activation. For example, in *C. crescentus*, DnaA-ATP is hydrolyzed by RIDA, but the resulting DnaA-ADP is then degraded by Lon protease (Wargachuk and Marczynski, 2015), and in *B. subtilis* and *S. aureus*, DnaA can rapidly exchange the bound ADP for ATP (Kurokawa et al., 2009; Bonilla and Grossman, 2012). Use of inhibitory proteins to block DnaA access to *oriC* binding sites would be equally effective for DnaA-ATP and DnaA-ADP. Known examples include CtrA in *C. crescentus* (Quon et al., 1998), AdpA in *Streptomyces* (Wolański et al., 2012), MtrA in *Mycobacteria* (Rajagopalan et al., 2010), and HP1021 in *Helicobacter* (Donczew et al., 2015). Topologically-sensitive DnaA binding sites identified in *H. pylori oriC* are an intriguing regulatory feature that would also be compatible with active DnaA-ATP or DnaA-ADP initiator, allowing DnaA to interact at some sites only when binding at other sites changes the origin’s superhelical density (Donczew et al., 2014). Anti-cooperativity factors are known to block DnaA-ATP oligomerization at some stage of orisome assembly. Versions include YabA (Merrikh and Grossman, 2011; Scholefield and Murray, 2013), SirA (Rahn-Lee et al., 2011), Soj (Scholefield et al., 2012), DnaD (Bonilla and Grossman, 2012; Scholefield and Murray, 2013), and Spo0A (Boonstra et al., 2013). While not yet identified, it is possible that factors may exist to block cooperative interaction between DnaA-ADP molecules (probably by blocking domain I interactions).

Some bacteria with the ability to assemble orisomes with a mixture of DnaA-ATP and DnaA-ADP may require cooperative binding in order to fill some of the DnaA binding sites, even those that are reported as consensus. For example, in the *Mycobacterium tuberculosis* origin, no individual R box can bind DnaA independently; rather cooperative binding between at least two recognition sites is required for the occupation the *oriC* (Zawilak-Pawlik et al., 2005). (It should be noted that *M. tuberculosis oriC* contains no R boxes with the 5′-TTATCCACA consensus sequence, so it is possible that none of the sites in *oriC*^*Mtb*^ have high enough affinity for DnaA to bind without cooperative interactions.) Formation of a bORC and progression to complete orisomes in this type of bacteria would require that DnaA recognition sites be closely spaced to allow interactions. This arrangement could be compatible with DnaA-ADP if domain I interactions were sufficient, but not all bacterial DnaAs can associate using domain I (Zawilak-Pawlik et al., 2017), and domain III interactions between DnaA-ATP molecules may be used exclusively. With an expanded view of DnaA-ADP activity, one can also envision origins in which DnaA-ATP is needed for the cooperative interactions used for site filling, but once DnaA-ATP is bound, ATP hydrolysis might provide a conformational change required for origin activation. The hydrolysis step could be intrinsic to the DnaA-ATP complex, or regulated by a factor analogous to the Hda protein in *E coli* (Katayama et al., 2017). Such a mechanism would explain why ATPase activity is required for complete orisome assembly in *M. tuberculosis* (Madiraju et al., 2006). In this scenario, DnaA-ATP would be required for origin recognition, but DnaA-ADP would perform the mechanical functions triggering initiation.

Under certain extreme conditions, such as the high temperatures, DnaA-ATP oligomers may be preferentially used to stabilize the orisome complex. The origins of these bacteria would have to contain closely spaced recognition sites to optimize the interaction between adjacent AAA + domains. Consistent with this idea, R boxes clustered in closely spaced arrays have been observed in the *oriC*s of the thermophilic bacteria *Thermus thermophilus* (Figure 4; Schaper et al., 2000) and *Aquifex aeolicus* (Erzberger et al., 2006). For these bacteria, DnaA-ATP oligomerization would be required for initiation, and it would be unlikely that functional orisomes would assembled using DnaA-ADP, even if that form could bind to the origin.

While the significance of any given arrangement of DnaA recognition sites remains speculative, regardless of the requirements for the ATP or ADP-bound forms, there is ample evidence that the configuration of every bacterial replication origin is optimized for its own DnaA, (Zawilak-Pawlik et al., 2005). For example, the DnaA proteins of both *E. coli* and *B. subtilis* bind with high affinities toward the same DnaA box sequence *in vitro* and create similar multimeric structures when visualized by EM (Krause et al., 1997). However, despite these apparent similarities, neither *E. coli* nor *B. subtilis* DnaA was able to unwind its heterologous partner origin. Similarly, while both *E. coli* and *M. tuberculosis* DnaAs bind well to *S. coelicolor oriC*, neither can bend the origin into the structure formed by the native DnaA protein (Jakimowicz et al., 2000; Zawilak-Pawlik et al., 2005). Further, heterologous *oriCs* replicate autonomously as plasmids or on the chromosome of another bacterial type only when their nucleotide sequences are nearly identical (Takeda et al., 1982; Zyskind et al., 1983; O’Neill and Bender, 1988; Roggenkamp, 2007; Demarre and Chattoraj, 2010). These data, combined with the many possible versions of *oriC* geography and accompanying regulation, make it difficult to determine whether there are features of orisome assembly widely shared by many bacterial orisomes. It is clear that more extensive analysis of different bacteria, as well as further analysis of synthetic origins, such as *oriC*^*allADP*^, will be necessary to reveal common paradigms for bacterial replication initiation and the specific roles of different DnaA forms.

## Author Contributions

All authors listed have made a substantial, direct and intellectual contribution to the work, and approved it for publication.

## Conflict of Interest Statement

The authors declare that the research was conducted in the absence of any commercial or financial relationships that could be construed as a potential conflict of interest.
